# Advances in Cellular Therapy for T-Cell Prolymphocytic Leukemia

**DOI:** 10.3389/fonc.2022.781479

**Published:** 2022-02-11

**Authors:** Indumathy Varadarajan, Karen Ballen

**Affiliations:** Department of Medicine, University of Virginia Cancer Center, Charlottesville, VA, United States

**Keywords:** T-PLL, CART cell, allogeneic stem cell transplant, CMV reactivation, autologous stem cell

## Abstract

T-cell prolymphocytic leukemia (T-PLL) is a rare, aggressive hematologic malignancy with a poor prognosis. Alemtuzumab (Campath) remains the cornerstone for treatment, with an 80% complete response (CR). Hematopoietic stem cell transplant (HSCT) is considered the standard of care as consolidative therapy in eligible patients. However, allogeneic stem cell transplant is also complicated by increased rates of infections from chemotherapy, acute graft-versus-host disease (GVHD), and chronic GVHD. This review aims to report the available literature on the efficacy and complications of consolidative HSCT. It also discusses the importance of patient selection and pre- and post-transplant complications including atypical infections and GVHD.

## Introduction

T-cell prolymphocytic leukemia (T-PLL) is a rare aggressive malignancy originating from the mature post-thymic T cell. Although the incidence of this malignancy is only 2.0/million/year in Western countries, it is considered as one of the most common mature T-cell leukemias (1). Patients usually present with a steep increase in lymphocyte counts, organomegaly, lymphadenopathy, and occasional skin lesions (2–4). Diagnosis is most often established by the presence of characteristic mature post-thymic T-cell immunophenotype on flow cytometry, that is, TdT−, CD1a−, CD2+, CD5+, and CD7+ positive (2). High expression of CD52 provides an effective therapeutic approach for these patients with Campath (alemtuzumab), an anti-CD52 monoclonal antibody that has robust activity in newly diagnosed and recurrent T-PLL (5, 6). Despite achieving impressive response rates of up to 80%, the median overall survival (OS) is only 10–16 months, as most patients relapse at 12 months. Very few options are available for salvage therapy after relapse (7, 8).

Single-gene sequencing has provided deep insight into the pathophysiology of this disease, thereby creating several potential therapeutic targets. Recent studies have discovered that the loss of ataxia telangiectasia mutated gene (ATM) and activation of T-cell leukemia/lymphoma gene play a pivotal role in oncogenesis (9). Targeted therapy with inhibition of HiDAC (Histone Deacetylase), BCL2 (B-Cell Lymphoma-2), and JAK-STAT (Janus Kinases, Signal Transducer and Activator of Transcription proteins) have shown to be very promising in Phase I and preclinical studies (9, 10). Despite multiple therapeutic options that are currently being studied, the current standard of care is a consolidative allogeneic stem cell transplant following induction therapy with Campath in transplant-eligible patients (11–13). Further collaborative studies combining these therapeutic modalities are needed to improve prognosis and OS.

## Role of Induction Agents in T-Cell Prolymphocytic Leukemia

Alemtuzumab remains the cornerstone agent for active T-PLL. It is a fully humanized anti-CD52 antibody that induces antibody-dependent cell lysis, apoptosis, and complement activation (14). Campath has shown overall responses (ORs) of up to 90% or higher when compared with traditional chemotherapy-based combinations (6, 7). Complete response (CR) rate at induction was not improved when used in combination with other conventional agents (15). In a pivotal study by Dearden et al., intravenous Campath resulted in an OR rate (ORR) of 91% and CR of 81%. These outcomes were superior to those of subcutaneous Campath, which showed a 33% CR, establishing intravenous Campath as the standard induction regimen (7, 8). Despite a high ORR, the duration of remission is short-lived, with most patients relapsing within 12 months, necessitating further consolidative therapy. Alemtuzumab can have a lasting impact, as its clearance decreases with repeated dosing, due to progressive loss of CD52 receptors from the destruction of malignant and normal T cells. This results in a 7-fold increase in concentration after 12 weeks of therapy (16). CD52 is a glycoprotein that is expressed on the cell surface of various hematopoietic cells. It is primarily expressed on the cell surface of mature lymphocytes, natural killer cells, eosinophils, neutrophils, monocytes macrophages, and dendritic cells (17). Hence, Campath treatment can have a lasting impact on the function of host and donor T cells, thereby influencing outcomes of consolidative transplant.

## Role of Hematopoietic Stem Cell Transplant

Hematopoietic stem cell transplant (HSCT) is an effective form of consolidation for T-PLL. Both autologous (Auto-HSCT) and allogeneic stem cell transplants (Allo-HSCT) prolong OS and progression-free survival (PFS) when compared with no consolidation therapy after induction Campath (11). Allogeneic stem cell transplantation is currently the only available potential curative option for T-PLL. Recommendation for consolidative stem cell transplant is primarily made from case reports and retrospective studies (11–13, 18–21).

## Consolidative Transplant Versus Observation

Krishnan et al. performed a multicenter retrospective analysis of 28 patients treated between 1996 and 2008 with either a consolidative autologous stem cell transplant (N = 15) or an allogeneic SCT (N = 13). OS and PFS were compared with those of 23 patients who were treated with Campath alone as first-line or second-line therapy. The patients in the non-transplant arm had achieved CR and survived for at least 6 months after the last dose of Campath. Among 15 patients who underwent autologous transplant, 11 patients were in CR1, 2 in CR2, and 2 in PR at the time of transplant.

All patients in this arm achieved a CR following an autologous transplant. Nine of these patients relapsed at a median of 15 months (5–56 months). There was 1 case of treatment-related mortality (TRM) secondary to pneumonitis. The median survival of patients receiving an autograft was 52 months. Among patients receiving allogeneic transplants, 9 were in CR1 and 4 in partial response (PR).

The allogeneic arm had 30% TRM that was attributed to fungal infection, refractory graft-versus-host disease (GVHD), pseudomonal sepsis, and Epstein–Barr virus (EBV)-associated post-transplant lymphoproliferative disorder (PTLD). Median OS was 33 months. The study showed a median OS of 48 months in patients receiving consolidative stem cell transplants (either auto or allo), which was more than the median survival in the non-transplant arm (20 months). The patients in the non-transplant arm were well-matched in patient characteristics to the transplant arm. This study showed that consolidation with HSCT after induction Campath was more beneficial than induction Campath alone. Even though patients had a median OS of 52 months with an Auto-HSCT and 33 months with an Allo-HSCT, the survival was not statistically different between these groups (p = 0.2). Patients undergoing allogeneic transplants had a high TRM of 30.7%, but survivors had long-term CR at a median follow-up of 6 years. The autologous arm, unfortunately, had a 60% relapse rate (RR), and all patients who relapsed died of progressive disease. This TRM may be reduced in the modern era with the introduction of reduced-intensity conditioning.

## Role of Allogeneic Stem Cell Transplant

Currently available recommendations are based on retrospective studies from international and national research organizations. There are a few prospective studies; however, no interventional study has been reported. Given the incidence of this disease, it would be very arduous to design such a study.

A retrospective study from the Center for International Blood and Marrow Transplant Research (CIBMTR) reported 47 patients who underwent an Allo-HSCT for PLL from 1995 through 2005; 77% of the patients received matched unrelated donors. Twelve patients in this group received partially matched or single allele mismatch. Median PFS at 1 year was 33% (95% CI of 20%–47%), and 1-year OS was 48% (95% CI of 33–62 months) with a median OS of 11.2 months. In this study, 46% of the patients had refractory PLL when they had an allogeneic stem cell transplant. Of the patients, 52% (95% CI 38–66) developed grade 2–4 GVHD, and the 1-year incidence of chronic GVHD was 42% (95% CI 28–57). Factors such as age, conditioning intensity, T- or B-PLL, CR after single or multiple lines of therapy (CR1 *vs.* CR2), and presence of acute or chronic GVHD were not shown to influence OS. Due to the size of the study and the heterogeneity in the patient population, the authors were unable to identify factors influencing outcomes with Allo-HSCT (12).

The European Society for Blood and Marrow Transplantation (EBMT) database has reported outcomes of 41 patients with T-PLL who underwent an allogeneic stem cell transplant from 1995 to 2006. Patients had received allografts from either a matched sibling donor (51%) or a matched unrelated donor. At a median follow-up of 36 months, this study reported a 3-year relapse-free survival of 19% and an OS of 21%. Three-year non-relapse mortality (NRM) and relapse incidence were 41%. Multivariate analysis showed that conditioning regimens containing total body irradiation (TBI) and a shorter interval between diagnosis and HSCT were associated with favorable relapse-free survival. No other recipient or donor-related factors had an impact on OS or PFS (13). Hence, this study further indicated that early referral to HSCT is associated with favorable outcomes.

The French registry reported a 36% (95% CI −17 to 54) 3-year OS and 26% PFS (95% CI 14–45) in 27 patients. Ten patients received HLA identical sibling allograft and 18 matched unrelated donors (one patient received a second Allo-SCT). Notably, this study only had 11% of patients who had refractory disease; the other patients were in complete remission or at least in a PR. With a median follow-up of 33 months, the estimated 3-year OS was 36% (95% CI −17 to 54%), and PFS was 26% (95% CI 14–45%). There were no factors associated with OS in the univariate analysis, and a trend for improved OS was seen in patients who received TBI in the conditioning regimen (21).

Most recently, EBMT has reported a prospective observational study of patients receiving an allogeneic stem cell transplantation for T-PLL from 2007 to 2012. A total of 54 patients were screened for this study. The study excluded patients with non-confirmed T-PLL diagnosis by a central laboratory, age ≥ 65 years, refractory disease at Allo-HSCT, cord, and mismatched unrelated donor transplants.

Thirty-seven patients were evaluable for the study endpoints; 44% of the patients received a transplant in CR1.

Most patients in the study had been treated with Campath before stem cell transplant, and the median time interval between the last dose of Campath and Allo-HSCT was 75 days; 30% of these patients received TBI doses of 6 Gy or higher. This study had a median follow-up of 50 months (12–78 months), the 4-year OS was 42% (25%–59%), and PFS was 30% (14%–46%). The median OS was 27.8 months, and PFS was 19.2 months. No factors were noted to have an impact on the outcome in multivariate analysis (22).

Single-center retrospective studies have reported a 4-year OS of 56%, NRM of 34%, a 4-year RR of 21%, a median PFS of 15 months (95% CI 12–99), and OS of 56 months [95% CI 15–56; (23)]. Sellner et al., in their case series of 10 patients, studied the utility of T-cell receptor (TCR)-based minimal residual disease (MRD) quantification for monitoring disease status in T-PLL. They reported a cumulative OS and PFS of 20%, an RR of 50%, and an NRM of 30% in the median follow-up period of 58 months (3–92 months). This interesting study aimed to correlate quantitative MRD monitoring by clone-specific real-time PCR of TCR rearrangements and the TCR repertoire diversity by next-generation sequencing (NGS). Patients who achieved MRD negativity with immunological interventions had a corresponding increase in the poly-clonality of their T cells (24).

**Table 1** summarizes the abovementioned studies and highlights important data including nature of transplant, disease status prior to transplant, OS, and TRM.

**Table 1:** Studies of Stem cell transplant for PLL.

Study	Auto- *vs.* Allo-SCT	Status at transplant	Conditioning regimen	Donor status	OS (median), months	Relapse rate	Acute GVHD grade 2–4	Chronic GVHD—1 year	Treatment-related mortality
Krishan et al.	Auto	CR1 and CR2, PR	84% TBI based		52	60% at 1 year	–	–	6.6%
	Allo	CR1, PR	MAC—33% All TBI based RIC—67% Flu/Mel	MUD 58% MRD—42%	33	30.7% at 1 year	23%	–	30.7%
Kalaycio et al.	Allo	CR, PR 46% refractory disease	MAC—40% >500 cGy or >9 mg/kg Bu RIC—30% <500 cGy or <9 mg/kg Bu Neither—30%	MRD—23% MUD—49% MMUD—25% Ukn—2%	11.2	39% at 1 year	52%	42%	28%
Wiktor-Jedrzejczak et al.	Allo	CR, PR 50% refractory disease	TBI based 54% Chemo based—32% Unknown—14%	MRD—51% MUD—49%	12	41% at 3 years	39%	44%	41% at 3 years
Guillaume et al.	Allo	CR, PR, 11% refractory disease	MAC—41% RIC—59% TBI based—56% Chemo—44%	MRD—37% MUD—63%	26	47% at 3 years	51%	40%	31% at 3 years
Wiktor-Jedrzejczak et al., 2019	Allo	CR, PR	MAC—35% (>6 Gy) RIC—65% (<6 Gy) Only TBI based	MRD—43% MUD—57%	27.8	38% at 4 years	19%	43%	32% at 4 years
Dholaria et al.	Allo	CR, PR	MAC—73% Flu/Bu Pen/Bu RIC—27% Flu, Cy TBI Flu, Mel	MRD—46% MUD—27% MMUD—18% Cord—9%	56	23% at 4 years	28%	54%	32% at 4 years
Sellner et al.	Allo	CR, PR	Flu Cy—40% Flu/TBI—60%	MRD—40% MUD—40% MMUD—10% Haplo-10%	10	50% at 58 months	–	–	30% at 58 months

Allo, allogeneic; Auto, autologous; CR1, complete response 1; CR2, complete response 2; PR, partial response; TBI, total body irradiation; MAC, myeloablative conditioning; RIC, reduced-intensity conditioning; Flu, fludarabine; Mel, melphalan; Bu, busulfan; Pen, pentostatin; Cy, cyclophosphamide; MRD, matched related donor; MUD, matched unrelated donor; MMUD, mismatched unrelated donor; Haplo, haplo-identical; Cord, cord blood; Ukn, Unknown.

Newly diagnosed T-PLL patients who need to be treated should be induced with intravenous Campath, preferably in experienced centers. All patients must be referred promptly to the Bone Marrow Transplant Team during induction. Based on the above-published retrospective studies, the National Comprehensive Cancer Network (NCCN) recommends that patients who obtain a CR or PR after initial therapy should be considered for a consolidative allogeneic stem cell transplant.

However, Allo-HSCT is associated with significant treatment-related mortality and morbidity. Patient’s performance status, donor availability, disease status at the time of HSCT, presence of atypical infections occurring secondary to Campath, and other general medical comorbidities play a crucial role in determining the risk versus benefit of proceeding with an allogeneic stem cell transplantation. The hematopoietic cell transplantation (HCT)-specific comorbidity index (HCT CI) published and validated by Sorror et al. includes a comprehensive pre-transplant assessment of preexisting comorbidities. A score of 3 or more in this assessment predicts 41% 2-year NRM (25). Autologous stem cell transplant as consolidative therapy can be considered in patients whose risk of undergoing an allogeneic stem cell transplant outweighs the potential benefit of cure. Although autologous stem cell transplant does not have the potential of cure, Krishna et al. reported an OS of 52 months in the Auto-SCT arm *vs.* 20 months in the non-transplant arm. Consolidative HSCT is preferred over observation after obtaining an optimal response to alemtuzumab. Prospective randomized trials with novel induction agents are crucially needed to improve outcomes; however, the rarity of this disease poses a significant challenge to the feasibility of such a study.

## Non-Relapse Mortality for Allogeneic Hematopoietic Stem Cell Transplantation

Most published data have reported 30%–40% treatment-related mortality; however, Allo-HSCT offers a potential long-term survival benefit for some patients. The main contributors to NRM are GVHD and infections. A retrospective analysis from the CIBMTR and EBMT did not show any association between age and mortality (12, 13). Recent advances in reduced-intensity conditioning regimens have reduced TRM in other diseases needing consolidative HSCT (26). Hence, it is hoped that the introduction of reduced-intensity conditioning regimens for T-PLL would result in improved TRM with longer follow-up.

In a single-center experience of treating more than 80 PLL patients, almost half of those achieving remission have proceeded to either an autologous or an allogeneic stem cell transplant. Most centers provide a “washout period” of 6 weeks to 3 months from completion of induction Campath to allogeneic stem cell transplant (Insert). This is thought to reduce the risk of failure of engraftment and reduce the risk of ongoing infection. In a case series reported by Shumilov et al., they noted that 5/10 patients succumbed to NRM. This was primarily attributed to post-transplant infectious complications. Cytomegalovirus (CMV) reactivation was observed in 60% of patients with 1 lethal infection. It is to be noted that no letermovir prophylaxis was given to these patients, and hence, the rates of reactivation may be lower in the letermovir era (27, 28).

Routine monitoring for CMV reactivation, anti-infective prophylaxis for herpes virus, and *Pneumocystis jiroveci* pneumonia are recommended for all patients even during induction with alemtuzumab-based regimens and must be continued during and post Allo-HSCT. These patients should be considered for letermovir prophylaxis if they have undetectable CMV DNA prior to transplant (28). It is advisable to screen these patients for fungal colonization with imaging and to consider further workup and treatment prior to stem cell therapy (29). Infectious screening for *Strongyloides* should be performed especially in patients originating from endemic regions, with the help of Serological testing and stool specimens. These patients can be treated with Ivermectin before transplant.

Screening for latent tuberculosis using QuantiFERON or Tuberculin skin test must be performed in these patients before stem cell transplant, and patients should be treated for latent tuberculosis infection (LTBI) concomitantly pre- and post-transplant (30).

Retrospective and prospective studies report an incidence of grade 2–4 acute GVHD ranging from 19% to 52%, with a 40%–55% incidence of chronic GVHD. The graft versus leukemia activity in T-PLL has been shown by correlating minimal residual kinetics (by TCR-based MRD quantification) with the TCR diversity alterations in patients receiving immunomodulation such as immunosuppression or donor lymphocyte infusions after an allogeneic transplant (24). Despite a washout period of 6 weeks from Campath, robust donor T-cell graft versus leukemia activity was noted in the study. Hence, early recognition and aggressive management of grade 2–4 GVHD play a pivotal role in improving treatment-related mortality. Therapeutic advancements and investigative trials in acute and chronic GVHD have led to the Food and Drug Administration (FDA) approval of agents like ruxolitinib, ibrutinib, and belumosudil (31–33). These recent advances should indeed contribute to decreasing treatment-related mortality in the upcoming years.

## Recent Advances in Cellular Therapy for T-Cell Prolymphocytic Leukemia

A recent case report has suggested acceptable toxicity to intrathecal (IT) Campath for refractory leptomeningeal prolymphocytic leukemia. IT Campath was also successful in the eradication of the leptomeningeal disease, which is resistant to triple IT chemotherapy and total brain irradiation (34). There are no published data on the efficacy of a consolidative allogeneic transplant in reducing the risk of central nervous system (CNS) relapse in T-PLL. CD30 is one of the cell surface proteins that is expressed on T cells, becoming an apt target against which chimeric antigen receptor-T (CAR-T) cells can be manufactured. However, targeting pan T-cell antigens not only would lead to severe T-cell immunosuppression but also would lead to autologous CAR-T destruction (35, 36).

CAR T-cell therapy has also been based on the TCR beta chain constant (TRBC) locus clonality; this technique may be more applicable in T-cell malignancies. Normal T-cell populations have a mixture of both TRBC 1- and TRBC 2-positive cells, while malignant T cells express only one beta chain. Hence, CAR T cells targeting the TRBC of the malignant clone would specifically target the malignant T-PLL cells and spare the normal T cells (37). The complementarity determining region 3 (CDR-3) is a hypervariable region of the TCR, which is responsible for binding the antigen. This would also be a potentially interesting target against which CAR T-cells can be manufactured (38).

There is a paucity of clinical trials for this uncommon disease. Several agents that have been implicated in the biology of T-PLL are currently being studied in phase 1 and preclinical studies. These include HiDAC, JAK-STAT, and BCL2 inhibitors (39–41). A combination of these novel agents with stem cell transplantation is also currently being studied in the form of post-transplant maintenance to reduce RRs (NCT02512497).

## Conclusion

1. Anti-CD52 antibody, Campath, as a single agent given intravenously remains the standard of care for induction therapy in T-PLL. Despite high ORRs, the CR is short-lived; and stem cell consolidation therapy is essential to provide an opportunity for cure (6).
2. Early referral to stem cell transplantation for patients receiving induction Campath is crucial for improving OS (13). All patients younger than 75 years should be referred for consideration of consolidative HSCT.
3. Allogeneic transplant is considered for patients who are younger than <75 years, with Eastern Cooperative Oncology Group (ECOG) <2, and with minimal comorbidities, as assessed by the HCT CI.
4. Response to Campath, availability of suitable donors, patient compliance, and adequate social support are some of the other important factors taken into consideration for patient’s suitability for Allo-HSCT.
5. Autologous HSCT can be considered in patients for whom the risk of an allogeneic transplant can outweigh the benefit, or in patients lacking suitable donors.
6. Adequate washout period of at least 6–12 weeks from Campath induction is preferred before proceeding with an allogeneic or autologous transplant.
7. Thorough investigation and treatment of underlying infections pre- and post-transplant play an important role in the reduction of mortality.
8. Reduced-intensity conditioning regimens, prophylactic antiviral agents such as letermovir, and the recent increase in the availability of multiple FDA-approved agents for acute and chronic GVHD are hoped to reduce TRM (26, 33).

This is an extremely exciting era for T-PLL, as deep insight into the intracellular mechanisms has led to the application of various agents to achieve an improved response.

The combination of these agents with cellular immunotherapy will elicit deep responses and improve RRs, thereby improving OS in this rare but fatal disease.

## Author Contributions

IV performed the literature search and data for the article. KB provided the concept and framework and edited and revised the article. All authors listed have made a substantial, direct, and intellectual contribution to the work and approved it for publication.

## Conflict of Interest

The authors declare that the research was conducted in the absence of any commercial or financial relationships that could be construed as a potential conflict of interest.

## Publisher’s Note

All claims expressed in this article are solely those of the authors and do not necessarily represent those of their affiliated organizations, or those of the publisher, the editors and the reviewers. Any product that may be evaluated in this article, or claim that may be made by its manufacturer, is not guaranteed or endorsed by the publisher.
